# Supplementary material for: Rare VPS35 A320V Variant in Taiwanese Parkinson’s Disease Indicates Disrupted CI-MPR Sorting and Impaired Mitochondrial Morphology
Source: Brain Sci. 2020 Oct 27;10(11):783. doi: 10.3390/brainsci10110783 (PMC7692537; doi:10.3390/brainsci10110783)

Supplemental Data:

Figure S1. Immunoblots of VPS35-ZsYellow1, CI-MPR, CTSD, SNCA and LAMP2A from three independent experiments.

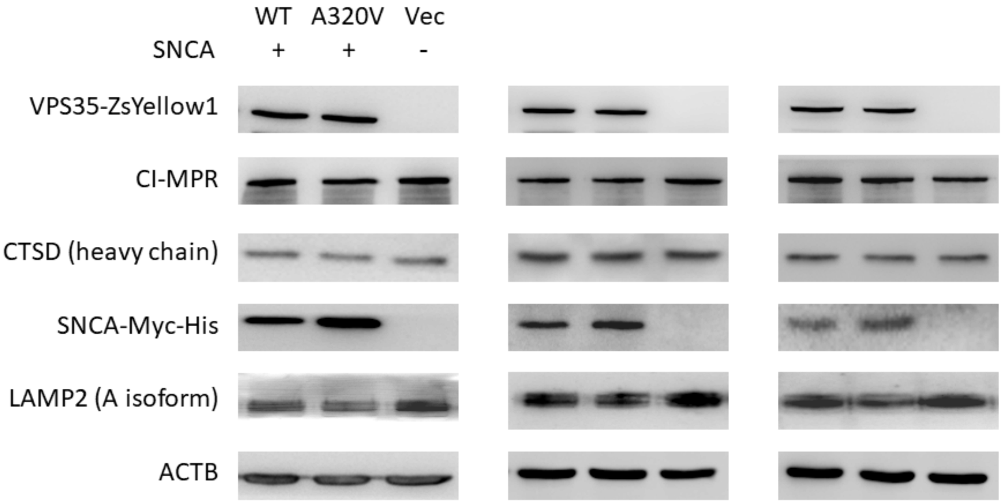

Figure S2. Immunoblots of VPS35-ZsYellow1, MUL1 and MFN2 from three independent experiments.

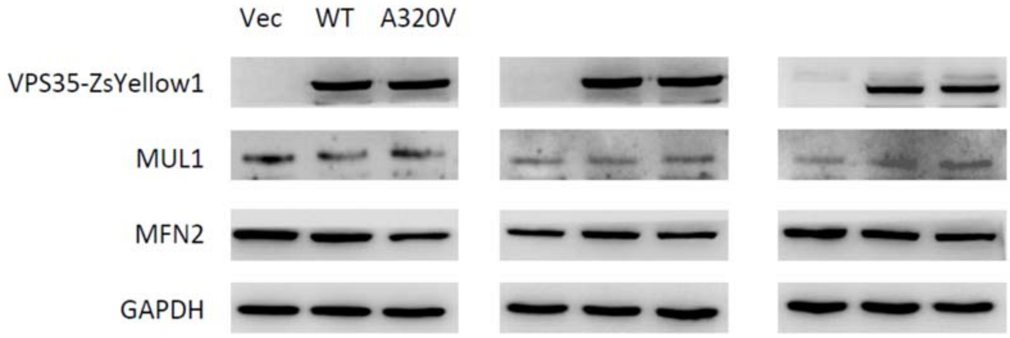

Supplement: Supplementary file 1 [file brainsci-10-00783-s001.pdf]
